# Missense Mutations of Codon 116 in the *SOD1* Gene Cause Rapid Progressive Familial ALS and Predict Short Viability With PMA Phenotype

**DOI:** 10.3389/fgene.2021.776831

**Published:** 2021-11-17

**Authors:** Xinmei Wen, Wenjia Zhu, Nan L. Xia, Qianwen Li, Li Di, Shu Zhang, Hai Chen, Yan Lu, Min Wang, Min Xu, Suobin Wang, Xin-Ming Shen, Jie Lu, Yuwei Da

**Affiliations:** ^1^ Department of Neurology, Xuanwu Hospital, Capital Medical University, Beijing, China; ^2^ Department of Rheumatology and Immunology, The Affiliated Drum Tower Hospital of Nanjing University Medical School, Nanjing, China; ^3^ Department of Radiology and Nuclear Medicine, Xuanwu Hospital, Capital Medical University, Beijing, China; ^4^ Beijing Key Laboratory of Magnetic Resonance Imaging and Brain Informatics, Beijing, China; ^5^ Department of Neurology and Neuromuscular Research Laboratory, Mayo Clinic, Rochester, MN, United States

**Keywords:** amyotrophic lateral sclerosis, SOD1, lower motor neuron, progressive muscular atrophy, rapid progression

## Abstract

Amyotrophic lateral sclerosis (ALS) is the most common form of motor neuron disease, characterized by a great variety of both clinical presentations and genetic causes. Previous studies had identified two different missense mutations in *SOD1* (p.R116C and p.R116G) causing familial ALS. In this study, we report a novel heterozygous missense mutation in the *SOD1* gene (p.R116S) in a family with inherited ALS manifested as fast-deteriorating pure lower motor neuron symptoms. The patient displayed similar clinical picture and prognostic value to previous reported cases with different R116 substitution mutations. Modeling of all R116 substitutions in the resolved SOD1 protein structure revealed a shared mechanism with destroyed hydrogen bonds between R116 and other two residues, which might lead to protein unfolding and oligomer formation, ultimately conferring neurotoxicity.

## Introduction

Amyotrophic lateral sclerosis (ALS) is a relentless neurodegenerative disorder and the most common adult-onset motor neuron disease (MND), which causes muscle weakness and atrophy. It is progressively fatal, and the majority of patients die within 3 years after onset of symptom (Turner et al., 2013). It is considered highly heterogeneous in nature, which displays a great variety of both clinical presentations and underlying pathogenic mechanisms. About 5%–10% of ALS cases are familial (fALS). The genetic spectrums of ALS vary among different populations. In European and American populations, the most common mutations are *C9orf72* intronic repeat expansions, whereas in Chinese and other Asian populations, *SOD1* mutations are most frequent (Zou et al., 2017; Liu et al., 2018). So far, more than 180 distinct *SOD1* mutations have been identified as genetic causes for ALS (Mathis et al., 2019) (https://alsod.ac.uk/). Mutant SOD1 proteins cause neurodegeneration mainly through a gain of toxicity pathway. Other possible mechanisms also include increased protein instability, protein aggregation, and probabilities of fibrillization (Wright et al., 2019).

The genotype–phenotype correlation has been described for a few *SOD1* mutations for disease durations but not for clinical features. p.A5V and p.G42S (previously denoted as p.A4V and p.G41S, respectively) *SOD1* substitutions have been consistently associated with a fast progressive phenotype, while patients with a p.H47R (previously denoted as p.H46R) mutation showed a more benign phenotype and a much longer life expectancy (Battistini et al., 2010; Takazawa et al., 2010). However, these mutations are marked by symptoms representing a varying degree of lower and/or upper motor neuron involvement of spinal, bulbar, and cortical regions. The genetic and phenotypic heterogeneities have been suggested to play critical roles in determining disease prognosis and in designing clinical trials (Manera et al., 2019). Whether there is a correlation between mutation status and clinical traits remains to be answered.

Here, we report a novel missense mutation in the *SOD1* gene (p.R116S), causing rapid deteriorating lower motor neuron symptoms in a fALS. The function of the mutant protein was explored to try to find a correlation between perturbed protein functions and clinical severity.

## Methods

### Pedigree

**FIGURE 1:**
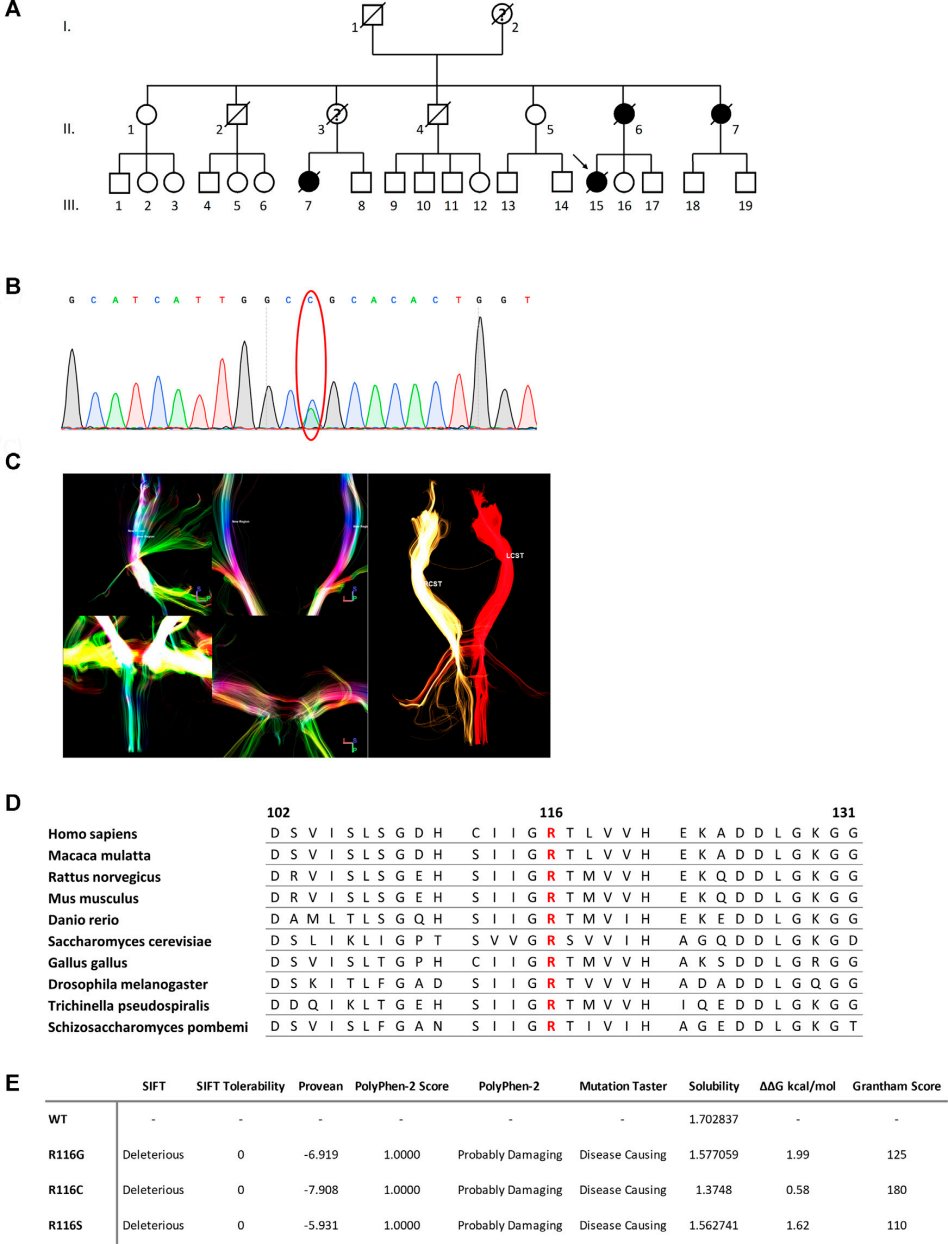
**(A)** Pedigree of the family. Square = male; circle = female; diagonal black line = deceased individual; black filled symbol = clinically proven affected individual; empty symbol = clinically healthy relative; question mark = suspected; arrow = proband. **(B)** DTI reconstruction of the bilateral cortical spinal tracts of the proband. RCST, right cortical spinal tract. LCST, left cortical spinal tract. **(C)** Confirmation by Sanger sequencing showing point mutation (red oval). **(D)** Sequence alignment analysis of SOD1 in different species. Amino acid at position 116 is highlighted in red. **(E)** Functional prediction of mutations at 116th amino acid in SOD1.

The proband was from a Chinese family of Han ethnicity with 28 known family members from three generations (Figure 1A). Overall, four affected family members have been diagnosed with MND. Two members from this family (proband and patient II-6) were seen at the ALS/MND Clinic, Xuanwu Hospital, Beijing, China, and further information on additional family members (II-7, III-7) was provided by the proband afterward. Disease onset was defined as first reported symptoms of weakness, dysarthria, or dysphagia. Patients underwent extensive laboratory studies to rule out other causes of neuropathy. The study was approved by the local medical ethical committee, with all participants providing written informed consent.

### Genetic Analysis

DNA was extracted from peripheral blood samples using a standard phenol–chloroform method. Whole-exome sequencing was used to detect gene mutations. DNA libraries were prepared with KAPA Library Preparation Kit and enriched using SeqCap hybridization probes following the manufacturer’s instructions (Roche, Basel, Switzerland). Captured DNA libraries were subsequently sequenced on the Illumina NovaSeq platform. Low-quality reads and adapter sequences were first excluded. Single-nucleotide variants (SNVs) and insertions and deletions (INDELs) were filtered using GATK. All called variants were annotated based on several public databases, including the 1,000 Genomes Project, Human Gene Mutation Database Professional, gnomAD, and China National Gene Bank (CNGB). The pathogenicity of the identified variants was analyzed mainly based on criteria following the American College of Medical Genetics and Genomics (ACMG) guidelines. Candidate mutation detected in exome sequencing was confirmed by Sanger sequencing.

### Protein Function Analysis and Structure Modeling

The sequence-based assessment of the potential pathogenicity of each variant was assessed by the SIFT web server (https://sift.bii.a-star.edu.sg), Provean Protein web server (http://provean.jcvi.org/genome_submit_2.php, PolyPhen-2 web server (http://genetics.bwh.harvard.edu/pph2/), CamSol web server (https://www-cohsoftware.ch.cam.ac.uk), and PopMuSiC web server (https://soft.dezyme.com). The protein crystal structure of the wild-type human SOD1 was retrieved from the Protein Data Bank repository (PDB code 2C9V), *in silico* mutagenesis was performed for the three mutations discussed in this study using Pymol (The PyMOL Molecular Graphics System, Schrödinger, LLC, http://www.pymol.org), and the Find function was used to identify potential hydrogen bonds.

## Results

### Clinical Findings

The proband was a 50-year-old female presented with a 2.5-month history of progressive muscle weakness. She first experienced a heavy feeling in her right leg when climbing stairs. It worsened over the next 2 weeks that she had trouble standing up from a squatting position without support. The weakness continued to progress and spread to her left leg in the following 2 weeks. By then, she noticed muscle wasting in her right thigh and experienced significant difficulty climbing stairs as well as trouble getting up from a sitting even with support. She soon showed problems reaching upward using her right arm. Neurological examination revealed mild to moderate weakness of proximal muscle groups in the lower limbs of both sides and the right upper limb. Muscle atrophy of the right thigh was noted. Deep tendon reflexes were decreased to normal with down-going plantar reflex on both sides. Other pathological reflexes were not elicited. There were no abnormal findings on sensory system examination. There were no cognitive or mood changes. Pulmonary function test indicated restrictive deficit. Electromyography showed in both upper and lower limbs as well as thoracic paraspinal muscles a neurogenic pattern. Sensory and motor nerve conduction velocities were within normal ranges. ^18^F-FDG PET did not detect any hypometabolism at the motor and premotor cortices or frontal–temporal regions. 3 Tesla magnetic resonance imaging (MRI) of the brain and spinal cord were unremarkable. Diffusion tensor imaging (DTI) reconstruction of the bilateral cortical–spinal tracts (CSTs) was performed, and no overt deficits were observed (Figure 1C). Together, a suspected diagnosis of progressive muscular atrophy (PMA) was made, and based on the familial history of MND, her blood sample was also sent out for WES and genetic analysis.

The proband received standard treatment with Edaravone and Riluzole and was evaluated every 3–6 months. Unfortunately, due to the COVID-19 pandemic, her follow-ups were only conducted *via* Telemedicine; therefore, detailed neurological check-ups were not possible. Her initial ALSFRS-R score was 39/48, which declined to 22/48 at her 3-month follow-up when she developed bulbar symptoms. Her ALSFRS-R score further decreased to 16/48 at her 5-month follow-up. One year after the disease onset, she was completely bedridden and required full-time non-invasive positive pressure ventilation (NIPVV). By then, her ALSFRS was 8/48. Six months later, she was admitted to the hospital and intubated due to respiratory failure and died 1 month after.

The proband’s mother (patient II-6) was diagnosed of MND at age 67 and died 1 year later. Her symptoms started with weakness and muscle wasting of the lower limbs. Later, she experienced weakness in upper limbs and complained of shortness of breath. The symptoms were more pronounced on the right side. Upon neurological examination, substantial proximal > distal bilateral muscle weakness of her lower limbs and mild weakness of her right upper limb were found. Atrophies of multiple muscle groups were also observed. Deep tendon reflexes were diminished as well. No pathological reflexes were detected, and both cranial nerves and sensory modalities were intact. There was no cerebellar sign. The pulmonary function test indicated restrictive deficit. Needle EMG showed chronic and active denervation in bulbar and spinal regions.

Patient III-7 was diagnosed of MND at age 52 and died of respiratory failure 1 year later. Her initial symptom included weakness in the upper limb. Another patient (II-7) in this family was also diagnosed of MND with lower-limb onset when she was 62 years old and died at age 63. Both underwent detailed laboratory studies that did not reveal any other cause of diffuse denervation. Both the mother (II-3) and the grandmother (I-2) of patient III-7 were suspected mutation carriers who died of other causes over the age of 70 years.

### Genetic Analysis

The DNA sample of patient III-15 was subjected to whole-exome sequencing (WES) to get a comprehensive profile of genetic variants. A novel heterozygous c.346C>A mutation (NM_000454.5, p.R116S, previously denoted as p.R115S) in the *SOD1* gene, was revealed (Figure 1B). Due to the death of all other affected members, segregation of the mutations could not be confirmed in the family presented here. However, this mutation was absent in the databases of healthy controls, including Genome Aggregation Database, the Exome Sequencing Project (ESP), and Exome Aggregation Consortium (ExAC) databases. The mutation altered a highly conserved residue and was predicted to be deleterious by both SIFT and PROVEAN and ranked as Probably Damaging and Disease Causing by PolyPhen-2 and Mutation Taster, respectively (Figure 1E).

### Prediction on Mutated Protein Function

*SOD1* R116S mutation-induced alterations in protein functions were explored using a battery of algorithms. Mutation at this site resulted in a polar to non-polar amino acid change, implying a significant change in amino acid chemistry (Grantham score of 110) and affecting a highly conserved residue (Figures 1D,E). The impact of R116S mutation on protein solubility was predicted using the CamSol Intrinsic web server, which suggested that the R116S SOD1 mutant was less soluble compared to the wild-type protein. Additionally, free energy change upon mutation was predicted using PopMuSiC, and an arginine-to-serine change at codon 116 was predicted to have a calculated ΔΔG of 1.62, suggesting a destabilizing effect.

**FIGURE 2:**
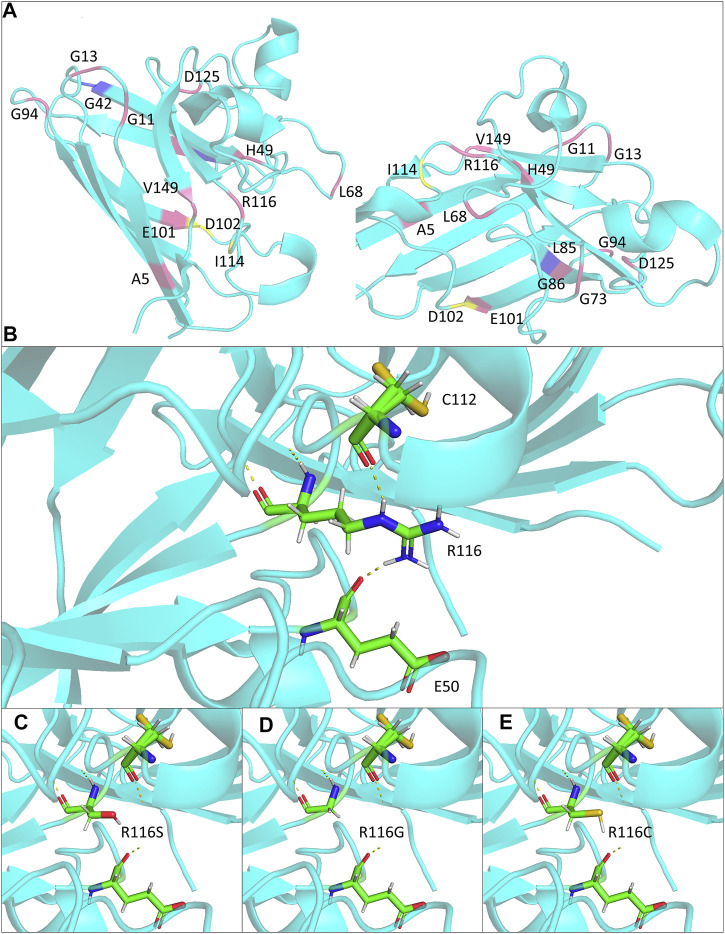
Structural modeling of the ALS-associated SOD1 R116 mutants in the study. **(A)** Monomer views of the SOD1 homodimer (PDB: 2c9v). Mutations discussed in Table 1 are highlighted and annotated. **(B–E)** Close lookup views of the hydrogen bonds (yellow dash line) for R116 and mutants R116S, R116G, and R116C, respectively.

The potential effects of the mutation on SOD1 protein stabilization were further investigated by protein modeling. SOD1 protein is a homodimer composed of two β-barrel monomers with copper and zinc in the active site. Codon 116 is located at the dimer contact site, being part of the interlocking Greek key loop connecting two four-stranded anti-parallel β-sheets facing each other (Figure 2A) (Wright et al., 2019). Interatomic interactions between the side chain of mutated residue and residues in the vicinity were analyzed, and hydrogen bonds were represented in the form of pseudo bonds using Pymol. Arginine at position 116 was shown to form hydrogen bonds with neighboring amino acids, particularly glutamic acid 50 and cysteine 112. R116S was found to disrupt hydrogen bonding with E50 and C112, suggesting alterations in native protein conformation and structure destabilization, thereby serving as a basis for neurotoxicity (Figures 2B,D).

## Discussion

We found a Chinese fALS family carrying a novel R116S change in *SOD1*. All four patients displayed an aggressive disease course with disease duration less than 2 years from initial symptom to death. Molecular modeling and bioinformatics analysis on mutant SOD1 protein suggested that loss of hydrogen bonds at the dimer interface, together with decreases in protein solubility and stability, which might explain the aggressive nature associated with the mutation.

The mean age at onset was 58 years in the family. The proband and her mother were characterized by a rapid disease progression with prominent lower motor neuron symptoms. Physical examinations showed no evidence of upper motor neuron signs, and there were no CSTs deficits on DTI in the proband, suggesting a progressive spinal muscular atrophy (PMA) phenotype of MND. It was suggested that around 10% of MND patients clinically presented as PMA (Van den Berg-Vos et al., 2009). Genetic causes might partially determine the motor neuron/clinical phenotypes. Some specific *SOD1* missense mutations have been associated with a consistent clinical presentation. Here, we summarized a group of *SOD1* mutations that have been found to cause a predominant lower motor neuron syndrome (Table 1). Most of the mutations belong to exon 4 and are located at β-strand structures of SOD1 protein (Figure 2A; Table 1). Regardless of the mutation status, these patients were more likely to have a limb onset with bulbar involvement during disease course and were also associated with a rapid disease progression. Consistent with clinical features, pathological findings showed severe spinal lower motor neuronal loss with minimal involvement of upper motor neurons of motor cortex and cortical spinal tracts (Table 1). Of notice, different missense mutations of the same site at the *SOD1* gene could lead to different disease phenotypes (i.e., A5V, A5T, previously denoted as A4V, A4T) (Table 1). It is reasonable to speculate that the relative position, property of the side chain of the mutated residue, and nature of the adjacent amino acid of the mutated residues were likely to affect the stability and conformation of SOD1 protein, thus conferring toxicity on mutant proteins.

**TABLE 1:** Clinical table of *SOD1* fALS patients with predominant lower motor neuron symptoms, color-coded by symptom group. The reference protein sequence for mutations included in the table is NP_000445.1.

Mutation	Exon	Structure	Site of onset		Bulbar sign	UMN sign	Weakness pattern		Disease course	CNS pathology	Primary references
A5V	1	β1-strand	Limb	Bulbar	Yes	Mild-absent	Distal	Bulbar	Rapid (1 ± 0.5 years)	Severe loss LMNs, absent to mild UMN abnormalities	Andersen et al. (1997); Cudkowicz et al. (1998)
A5T	1	β1-strand	Limb		Yes	Mild-absent	Proximal		Rapid	Severe LMN loss, mild CST damage, no UMN loss	(Takahashi et al., 1994; Aksoy et al., 2003)
G11V	1	β1-strand	Limb	Bulbar	Yes	Mild-absent	Distal	Bulbar	Rapid (14.7 ± 5.3 m)	N/A	Kim et al. (2007)
G13R	1	Loop I	Limb		Yes	Absent	Distal		Slow	N/A	Penco et al. (1999)
G42S^a^	2	β4-strand	Limb		Absent	Absent	N/A		Rapid (<15 m)	N/A	Subramony et al. (2011)
			Limb		Yes	Mild	N/A		Rapid (11.6 ± 1.7 m)	N/A	Rainero et al. (1994)
H49G	2	β4-strand	N/A		N/A	Absent	N/A		Rapid (8 m)	N/A	Enayat et al. (1995)
L68P	3	Loop IV	Limb		Absent	Absent	Distal		Slow	N/A	del Grande et al. (2011)
G73C	3	Loop IV	Limb		Yes	Absent	Proximal		Slow (53 m)	N/A	Stewart et al. (2006)
L85V^a^	4	β5-strand	Limb		Yes	Absent	Distal		Rapid (<1.5 years)	N/A	Aoki et al. (1995)
			Limb		Yes	Mild	N/A		Moderate (4.8 ± 2.7 years)	N/A	Ceroni et al. (1999)
G86S	4	β5-strand	Limb		Absent	Absent	Distal		Rapid (15–18 m)	N/A	Takazawa et al. (2010)
G94C	4	Loop V	Limb		Absent	Absent	Distal		Slow (153.0 ± 46.1 m)	Severe LMN loss, minimal involvement of CST	Regal et al. (2006)
E101K	4	β6-strand	Limb		Yes	Absent	N/A		Slow (10-20 years)	TDP-43 negative neuropathology	Rabe et al. (2010)
D102N^b^	4	β6-strand	Limb		Mild	Absent	Distal		Rapid (∼28 m)	Severe loss of LMNs, relatively well preserved UMNs, undamaged pyramidal tracts	Cervenakova et al. (2000)
I114T^b^	4	Loop VI	Limb		N/A	Absent	Distal		Rapid (∼3 years)	Severe LMN loss, well preserved UMNs and CSTs	Rouleau et al. (1996)
R116G	4	β7-strand	Limb		Yes	Absent	Proximal		Rapid (2-3 years)	N/A	Rabe et al. (2010)
R116S	4	β7-strand	Limb		Yes	Absent	Proximal		Rapid (<2 years)	N/A	our study
D125G	5	Loop VII	Limb		Yes	Absent	Proximal		Moderate (2–5 years)	N/A	Ricci et al. (2019)
V149I	5	β8-strand	Limb	Bulbar	Yes	Absent	Distal	Bulbar	Rapid (1.8 ± 0.5 years)	N/A	Abe et al. (1996)

a The reported mutation has been reported to show predominant LMN symptoms but with different accompanying symptoms in separate studies.

b The reported mutation has been reported to manifest as classical ALS phenotype in separate studies.

N/A, not available.

To our knowledge, the R116S mutation has never been reported so far (http://alsod.iop.kcl.ac.uk). However, R116G was found to be the most common *SOD1* mutation in German fALS patients, which accounts for up to 44% of *SOD1* associated ALS patients and was suggested to arise from a common founder (Niemann et al., 2004; Rabe et al., 2010). A different point mutation R116C was identified in one Italian sporadic ALS patient (Tortelli et al., 2013). Consistently, these two mutations are also associated with rapid disease progression and predominant lower motor neuron symptoms (Table 2). R116 mutations of *SOD1* in three distinct ethnic groups caused the similar severe phenotype, indicating that R116 is critical for the protein function. Additionally, it has been suggested that protein aggregation is favored by mutations that bring the net charge of the protein closer to neutrality (Lindberg et al., 2005). Thus, the substitution of a positively charged amino acid (arginine) with a neutral one (glycine, cysteine, or serine) would probably promote protein aggregation and lead to neurotoxicity.

**TABLE 2:** Clinical features of previously reported patients carrying *SOD1* mutations of R116. The reference cDNA and protein sequences for mutations included in the table are NM_000454.5 and NP_000445.1.

Family/Individual	Family history	Ethnicity	Patient I.D.	Mutation cDNA (NM_000454.5)	Amino acid change (NP_000445.1)	Gender	Age of onset	Age of death	Avg. age of onset	Site of onset	Bulbar symptom	Proximal vs. distal	Disease duration	Affected members	Generations (n)	References
1	fALS	German	Mother	c.346C>G	p.R116G	F	66	68	60	LL	Y	Proximal	2-3 y	6	3	Rabe et al. (2010)
			Son			M	43	45			Y	Proximal				
2	fALS	German	N/A	c.346C>G	p.R116G	N/A	67	N/A		LL	Y	Proximal		2	N/A	Rabe et al. (2010)
3	fALS	German	N/A	c.346C>G	p.R116G	N/A	64	N/A		LL	Y	Proximal		2	N/A	Rabe et al. (2010)
4	fALS	German	III-ALS3.7	c.346C>G	p.R116G	N/A	42	N/A		LL	Y	Proximal		5	3	Kostrzewa et al. (1994); Niemann et al. (2004); Rabe et al., 2010
			II-2			F	66	68								
			II-3			F	48	51								
5	fALS	German	ALS73.1	c.346C>G	p.R116G	N/A	52	N/A		LL	Y	Proximal		2	5	Niemann et al. (2004)
6	fALS	German	ALS129.1	c.346C>G	p.R116G	N/A	60	N/A		LL	Y	Proximal		5	4	Niemann et al. (2004)
7	fALS	German	ALS179.1	c.346C>G	p.R116G	N/A	74	N/A		LL	Y	Proximal		3	3	Niemann et al. (2004)
8	fALS	German	N/A	c.346C>G	p.R116G	N/A	56	N/A		LL	Y	Proximal		3	N/A	Rabe et al. (2010)
9	fALS	German	N/A	c.346C>G	p.R116G	N/A	N/A	N/A	N/A	N/A	N/A	N/A	N/A	N/A	N/A	Muller et al. (2018)
10	fALS	Chinese	III-15	c.346C>A	p.R116S	F	50	51	51	LL	Y	Proximal	20 mon	4	2	our study
			III-7			F	52	53		UL	Y		<1 y			
			II-6			F	67	68	65	LL	Y		14 mon			
			II-7			F	63	64		LL	Y		<1 y			
11	sALS	Italian	N/A	c.346C>T	p.R116C	M	72	N/A	N/A	UL	Y	N/A	>2 y	N/A	N/A	Tortelli et al. (2013)
12	N/A	Japanese	patient 21	c.346C>G	p.R116G	M	66	N/A	N/A	LL	N/A	N/A	>22 months	N/A	N/A	Sato et al. (2005)
13	N/A	American	N/A	N/A	p.R116G	N/A	N/A	N/A	N/A	N/A	N/A	N/A	N/A	N/A	N/A	Andersen et al. (2003)

LL, lower limb; UL, upper limb; N/A, not available; Y, yes.

Within the resolved protein structure of SOD1, R116 forms hydrogen bonds with E50 and C112, respectively, both of which were reported to play important roles in SOD1 protein stability and function. Specifically, E50 locates in a loop structure, whose alterations could cause β-sheet partial unfolding, rendering it more prone to aggregation (Ding and Dokholyan, 2008). On the other hand, C112 is a primary target for redox attacks, the oxidation of which results in conformational changes of SOD1 protein and formation of oligomers, ultimately leading to axon transport deficits and motor neuron death (Bosco et al., 2010; Nagano et al., 2015). Notably, all R116 substitution mutations reported to date, including R116S, R116C, and R116G, are predicted to abolish the two hydrogen bonds and likely to affect the relative positions of E50 and C112 (Figures 2C–E), which in turn might cause β-sheet partial unfolding and might make C112 more accessible for oxidation, leading to mutant SOD1 misfolding and toxicity.

Taken together, we report a *SOD1* mutation site causing the most consistent phenotype: ubiquitous limb onset characterized by prominent LMN presentation with rapid progression, adding to the list of ALS causing mutations bearing a genotype–phenotype correlation. Additionally, it displays a prognostic value of *SOD1* R116 missense mutation.

## Data Availability

The datasets for this article are not publicly available due to concerns regarding participant/patient anonymity. Requests to access the datasets should be directed to the corresponding author.
